# Institutional Residents and Recreation

**Published:** 1914-06-13

**Authors:** 


### INSTITUTIONAL RESIDENTS AND RECREATION.

### The Escape from the Common-Room Atmosphere.

It is always comforting to find oneself desirous of being delivered of a platitude. The number of "contrary" things to be said in this philistine country is so great that when the proposition to be advanced is an acceptable one, when for once the strain of balancing between insincerity and discourtesy is blessedly absent, then audience and speaker both are entitled to a little self-congratulation. The proposition we have in mind just now is that the institutional resident is of all men and women the one who needs especially recreation. And as obviously, the reason is that the institutional resident has no domestic castle, not of indolence, but of privacy, to retire to. Let social reformers preach and laugh as much as they will, let them bring forward again and again Dr. Archdall Reid's dictum that national traits are traditional and not congenital, all the same the fact persists that people here like to rest from their labours somewhere else than upon the scene of them. They can adapt themselves to the opposite condition of things, of course, as some of our readers well know; but it is at the price of a steady little drain of effort and of nerve energy, and then of amiability. The Frenchman who moves with daily fatality to a café, the German who haunts smoky restaurants, might not get the *ablehnend* feeling that the continual atmosphere of the common-room sometimes gives an Englishman. In the last case, the fact of recreation being an escape from this atmosphere is the reason, just as much as its forming an occupational change, why our institutional residents' nervous mechanisms automatically formulate within them a demand for it.

During the winter the playhouse, with its one sightworthy play in twenty, will have given some relief. But now spring has become more than a mere indication, rural scenery, the unaccustomed dose of fresh air that sends one home sleepy at night, that scenery whose beauty it is so hard to realise really lies in the eyes of us beholders, will be the thing craved for. And for all weary people it is mechanically reproduced and recalled very well in the pages of our contemporary, *Country Life*, in a recent number of which is given an illustration showing one of the old-time buildings still numerous in the town of Coventry. Before the door—contrast is an artifice always dear to the artist—stands a wire-wheeled limousine, probably also a local product, which clearly belongs to the teens of the twentieth century. But it did not need that to show that Ford's Hospital, with its one picturesque storey, is a relic, a still useful one, of the past. We read that "thanks to the generosity of the early merchant stapler who founded this charitable institution, it houses in comfort fifteen aged women." That, of course, is more often than not the connotation which a hundred years ago was attached to the word "hospital." What corresponded to our present hospitals were called infirmaries. Even as late as Anthony Trollope's time, we read of a "hospital" furnishing one of the clerical characters of "Barchester Towers" with a good living and nominal duties, the other beneficiaries being a handful of old people past work but not invalids, who seemed to wear a chastened, pietistic air as of necessity. Well, all such administrative abuses must have been amended by now. Long may these old buildings remain to delight the eye and the age-reverencing instinct, even though that were to be a psychosis! One can imagine a companion picture, but it would not be so pleasing—a carrier's cart at the main entrance of some huge, smart, enchantment-lacking creation of the Metropolitan Asylums Board.

